# The Promises and Pitfalls of Retrieval-Extinction Procedures in Preventing Relapse to Drug Seeking

**DOI:** 10.3389/fpsyt.2013.00014

**Published:** 2013-03-12

**Authors:** Kate Hutton-Bedbrook, Gavan P. McNally

**Affiliations:** ^1^School of Psychology, The University of New South WalesSydney, NSW, Australia

**Keywords:** addiction, reinstatement, relapse, reconsolidation, memory, extinction

## Abstract

Relapse to drug seeking after treatment or a period of abstinence remains a fundamental challenge for drug users. The retrieval – extinction procedure offers promise in augmenting the efficacy of exposure based treatment for drug use and for protecting against relapse to drug seeking. Preceding extinction training with a brief retrieval or reminder trial, retrieval – extinction training, has been shown to reduce reinstatement of extinguished drug seeking in animal models and also to produce profound and long lasting decrements in cue-induced craving in human heroin users. However, the mechanisms that mediate these effects of retrieval – extinction training are unclear. Moreover, under some circumstances, the retrieval – extinction procedure can significantly increase vulnerability to reinstatement in animal models.

Drug addiction involves the compulsive use of drugs despite adverse consequences (Torregrossa and Taylor, 2012). It imposes significant burdens on the individual drug user, their families, and communities. The successful treatment of drug users not only improves the health and well being of the user, but brings significant economic benefit to the broader community via reductions in criminal activity as well as reductions in health services utilization (McCollister and French, 2003). However, the fundamental problem with existing treatments for drug addiction is that they are ineffective at promoting long-term abstinence. The vast majority of drug users will relapse to drug use in the first year following treatment or abstinence (Hunt et al., 1971; Heinz et al., 2008). Relapse is elicited by a number of factors such as stress and negative affect (Shiffman and Waters, 2004), and exposure to drug-related places, people, and cues (Drummond et al., 1995). Unsurprisingly, many treatments have attempted to reduce the power of these factors over drug taking by implementing cue-exposure protocols (Heather and Bradley, 1990; Hammersley, 1992). Typically these treatments involve the non-reinforced exposure to drug-related stimuli and the drugs themselves. For example, the smoker may be exposed to the sight and smell of a burning cigarette, the heroin user to the sight and feel of a loaded syringe and tourniquet. Yet, although these treatments can be successful in reducing responding elicited by such stimuli in the short-term, they yield at best extremely modest long-term efficacy (Conklin and Tiffany, 2002).

In animal models of drug taking, extinction training also produces short-term decrements in drug seeking without long-term protection from reinstatement. Rats, for example, readily learn to self-administer a variety of drugs abused by humans. Drug seeking behavior can be extinguished when the contingency between drug seeking and delivery of the drug reward is broken. However, drug seeking is not permanently lost following extinction. Drug seeking can be reinstated under a number of conditions including following presentations of a drug prime (De Wit and Stewart, 1981), a drug associated stimulus (Davis and Smith, 1976; De Wit and Stewart, 1981), or by a return to the training context when extinction training occurs in a different context (Crombag and Shaham, 2002). In each of these experiments, extinction was achieved by omitting the drug reinforcer as well as any drug associated stimuli. The finding that responding which has been lost via extinction training can be recovered or restored under these different conditions has been interpreted to mean that extinction training does not erase or over write the original drug seeking memory. Rather, extinction training is believed to result in formation of a new memory. This extinction memory competes with the drug seeking memory for expression and for control over motivation and behavior. Specifically, the extinction memory is context-dependent, so that extinction is retrieved, and drug seeking inhibited, only under conditions similar (e.g., context, time) to extinction training (Bouton, 2000).

Due to the apparent failure of standard extinction training to yield long-term behavioral change in humans and other animals, a growing body of literature has begun to focus on the processes of consolidation and reconsolidation of memories in order to promote a permanent change in the original memory and hence a permanent change in behavior. Reconsolidation refers to the process by which a retrieved memory enters into a labile state that requires *de novo* protein synthesis to be “reconsolidated” back into a stable long-term memory. During this labile or active state, that may last as long as 6 h (Nader et al., 2000), the memory is unstable and may be altered, for example to incorporate new information and/or alter its original contents. It is possible to disrupt the memory during this state with pharmacological agents that interfere with the protein synthesis or other cell biological processes required for reconsolidation. For example, pharmacological manipulations may inhibit the reconsolidation of a drug stimulus memory and thereby prevent that stimulus from controlling behavior on later presentations (Lee et al., 2006; Milton et al., 2008). While this approach has provided insights into the molecular mechanisms that underlie memory reconsolidation, there are a number of limitations with translating this approach to a human clinical population. Most importantly, many of these compounds are toxic or have not been approved for human clinical use. Recently, however, a new non-pharmacological approach has been developed that appears to circumvent many of these limitations to human application.

## Retrieval – Extinction Procedures

The first evidence for a non-pharmacological disruption of reconsolidation, a “memory retrieval-extinction” procedure, was provided in an animal model of fear, in which a single reactivation trial provided prior to an extinction session prevented later recovery of this fear memory (Monfils et al., 2009). Rats were trained to fear a tone conditioned stimulus (CS) via pairings with a shock unconditioned stimulus (US). The following day the animals were presented with a brief (one tone CS) “reminder” cue followed 10 min, 1, 6, or 24 h later by extinction training that involved a further 18 CS alone presentations; 24 h later the animals were tested for long-term memory and following this for either a renewal or spontaneous recovery test. Rats in both groups showed normal loss of fear during extinction training. Rats that received standard extinction training also showed the normal reinstatement of fear via tests of renewal and spontaneous recovery. The rats that received the retrieval + extinction training did not show any recovery of fear. This retrieval – extinction training prevented the recovery of fear in this model. Retrieval – extinction training also produces relatively permanent fear loss in humans. In normal human subjects, Schiller et al. (2010) reported that a retrieval-extinction procedure rendered experimentally acquired fear resistant to reinstatement and spontaneous recovery. While these findings provide some evidence that the behavioral disruption of reconsolidation may reduce recovery of extinguished fear, it is important to note that there have been some successes (Clem and Huganir, 2010; Rao-Ruiz et al., 2011) and some failures (Chan et al., 2010; Costanzi et al., 2011; Soeter and Kindt, 2011) in replicating these findings.

Recently, Xue et al. (2012) adapted this retrieval – extinction protocol to study its effect on drug seeking in both non-human and human populations. For example, Xue et al., trained rats to self-administer intravenous heroin for 3 h/day for 10 days. The rats readily learned to do so. Then, during extinction, a normal extinction group received 14 daily 195 min extinction sessions whereby responses no longer yielded the drug reward. A Retrieval – extinction group also received 14 daily sessions but these were divided into a 15-min retrieval session followed 10 min later by a longer 180 min extinction session. In both these daily sessions, responding was not reinforced. Both groups showed the normal decline in heroin seeking across the course of extinction training. Later when tested for heroin priming reinstatement, the normal extinction group showed robust reinstatement whereas the retrieval – extinction group did not. Xue et al., were able to report similar effects for the cocaine primed reinstatement of cocaine seeking and spontaneous recovery as well as context-induced reinstatement of cocaine seeking. The effectiveness of the retrieval-extinction procedure in preventing reinstatement has also been shown in an animal model of alcohol seeking. Millan et al. (2013) trained rats to respond for alcoholic beer. They then extinguished this responding. Whereas rats subjected to normal daily 1 h extinction training sessions later showed a robust context-induced reinstatement of alcohol seeking, rats that had received a 10-min retrieval session prior to a 50-min extinction session did not.

Remarkably, Xue et al. (2012) were able to extend these findings to cue-exposure treatments of heroin addicts in an inpatient treatment setting. On Day 1, participants rated craving levels following exposure to a 5-min video consisting of heroin cues. On Days 2 and 3, the participants were exposed to a 5-min video of heroin cues followed by extinction of these cues 10 min or 6 h later. Blood pressure and heart rate were monitored before and after cue-exposure. In this experiment, normal extinction training (i.e., neutral video followed by heroin cue extinction) produced no significant reduction in cue-induced craving or blood pressure changes. In contrast, the retrieval + extinction group (heroin video followed by heroin cue extinction) showed significant reductions in cue-elicited craving and blood pressure changes. These reductions were also long lasting, persisting up to 6 months following the brief 2 day extinction protocol. It remains to be determined whether the protective effects of this retrieval – extinction manipulation generalize beyond the treatment setting.

## Not Memory Erasure and Not Always Protective

The effects of the retrieval-extinction procedure on extinction of drug seeking have been interpreted as a behavioral disruption of the reconsolidation process (Monfils et al., 2009; Schiller and Phelps, 2011; Milton and Everitt, 2012). This is based on the assumption that standard extinction training yields new memory formation that competes with rather than replaces the original memory (Bouton, 1994). When extinction occurs following a retrieval trial, the original memory is assumed to be destabilized and labile allowing the extinction training to directly modify the original memory (Monfils et al., 2009; Torregrossa and Taylor, 2012). According to this interpretation, retrieval-extinction training leads to a change in the original memory that prevents the original memory from supporting reinstatement of drug seeking. Leaving aside the difficulties with making inferences based on the absence of responding (Lattal and Wood, 2013), reconsolidation theory yields two clear predictions about the process and mechanism underlying retrieval-extinction manipulations.

First, a key prediction of reconsolidation theory is that for the retrieval – extinction procedure to be successful, extinction training must occur inside the “reconsolidation window” (Monfils et al., 2009). The reconsolidation window is the hypothetical period of time after memory retrieval during which the memory is destabilized and yet to be reconsolidated. It is this period of destabilization that is purported to enable extinction training to directly modify the original training memory. The evidence in support of this comes from experiments that have shown that extinction training conducted outside the reconsolidation window is ineffective at preventing later reinstatement. For example, Xue et al. (2012) reported that if retrieval preceded extinction training by 6 h in either humans or rats, then it was ineffective at preventing reinstatement. Thus, according to reconsolidation theory, the brief retrieval session must occur prior to extinction in order to disrupt the reconsolidation process. Millan et al. (2013) tested this possibility. Rats were trained to respond for alcoholic beer in daily 1 h sessions. Then responding was extinguished in daily sessions. For the control group, extinction consisted of daily 1 h sessions. For the retrieval – extinction group, extinction consisted of daily 50 min sessions followed 70 min later by a 10-min retrieval session. Recall that Millan et al. (2013) showed previously that the daily 10 min then 50 min sessions (i.e., retrieval + extinction training) yielded a resistance to reinstatement. In this experiment, a reversed extinction + retrieval manipulation likewise yielded a resistance to reinstatement of alcoholic beer seeking. This finding is opposite to that predicted by reconsolidation theory. Reconsolidation theory predicts that the retrieval trial must occur before extinction training in order to reactivate the original memory and allow the new extinction learning to be incorporated prior to reconsolidation (Tronson and Taylor, 2007; Nader and Hardt, 2009; Schiller and Phelps, 2011). It is not possible within this theory for a retrieval trial to act retrospectively on encoding of the extinction memory.

A second key prediction of reconsolidation theory is that the disruption of reconsolidation should be protective. The retrieval – extinction procedure, by directly targeting the original drug taking memories, removes, or severely weakens the basis for reinstatement and so should always protect against reinstatement in animal models and relapse in humans. According to the theory, this manipulation is not only protective but in fact, because it is held to directly alter the original drug seeking memory, it returns the animals to a state similar to that of a naive animal. The available evidence is partly consistent with this. The retrieval – extinction procedure is effective in reducing or abolishing reinstatement across a variety of forms of reinstatement in animal models including spontaneous recovery, drug priming reinstatement, and context-induced reinstatement. However, these forms of reinstatement fail to adequately model a key feature of relapse to drug taking in humans. Such relapse involves drug seeking behavior that yields a drug reward. In the animal models of reinstatement, the drug reward is not available on test. Millan et al. (2013) examined whether the retrieval-extinction procedure would likewise protect animals against reinstatement when the drug reward was contingently available on test. In this experiment rats were trained to respond for alcoholic beer. This responding was then extinguished. For the normal extinction group, extinction training consisted of daily 1 h extinction sessions. For the retrieval – extinction group, extinction training consisted of daily 10 min then 50 min extinction sessions separated by 70 min. Both groups were then tested under a progressive ratio (PR) schedule of reinforcement. The PR test is a widely used measure of the motivation to respond for and consume drug rewards. Importantly, Millan et al. (2013) included a third group on test that had never been trained or extinguished before. This naive group allowed assessment of the possibility that the retrieval – extinction manipulation rendered animals similar to drug naive animals. The PR tests showed that both the normal extinction and retrieval – extinction groups were more motivated to respond for the drug reward than the naive group. Hence, retrieval – extinction training did not return animals to a state similar to a naive animal. Moreover, these tests showed that the retrieval – extinction manipulation significantly increased the motivation of animals to respond for and consume the drug relative to standard extinction training. These testing conditions model a key feature of relapse to human drug taking. This finding is theoretically interesting because it suggests boundary conditions on the effectiveness of retrieval – extinction training in protecting from reinstatement and it helps identify the precise mechanism of this training. It is practically significant because it may suggest caution in the application of the retrieval – extinction procedure to clinical settings. At minimum, it draws attention within the neuroscience field to the well known clinical possibility that the factors promoting or hindering a lapse may be different to those promoting or hindering relapse to drug taking (Marlatt et al., 1988). These findings were similar to those reported by Ma et al. (2011), where reinstatement of a previously extinguished CPP was augmented in a test 4 weeks after retrieval – extinction training. Taken together, these results suggest that the retrieval-extinction procedure is not always protective against reinstatement and, under some conditions, may actually increase vulnerability to reinstatement.

## Beyond Reconsolidation: Understanding How Modified Extinction Training Protocols Yield Long Lasting Behavior Change

Given the profound health, medical, and economic impact of drug use, there is a clear need for new approaches that effectively undermine the persistent propensity of drug users to relapse to drug taking after a period of abstinence and/or extinction. Under some circumstances, retrieval – extinction procedures can produce longer lasting behavioral change than a standard extinction procedure. This extends across a variety of drug reinforcers (heroin, cocaine, alcohol) and different self-administration procedures. Importantly, the protective effects of this retrieval-extinction procedure extend to studies of cravings in human drug users. This generalizability across drug classes and species, as well the procedural simplicity of the retrieval – extinction training, marks the retrieval-extinction procedure as an exciting and promising technique for experimental investigation and therapeutic intervention.

However, at the same time, this technique is poorly understood. The findings reviewed here question both the cause and the consequences of the retrieval – extinction protocol. The finding that a reversed extinction – retrieval manipulation is effective at attenuating some forms of reinstatement is inconsistent with the possibility that this is a behavioral disruption of reconsolidation. The finding that retrieval-extinction may increase vulnerability to reinstatement when testing conditions involves contingent presentations of the reinforcer shows that the retrieval – extinction procedure is not always protective. It is possible that this procedure deepens the learning that normally happens during extinction. Consistent with this is the finding that retrieval – extinction training potentiated extinction-induced changes in PKM**ζ** expression in the amygdala and prefrontal cortex (Xue et al., 2012) and deepened extinction learning can augment resistance to reinstatement (Janak and Corbit, 2011). However, a deepened extinction explanation has difficulty explaining the augmented responding during tests of reacquisition. It is important that the mechanisms for retrieval-extinction training be further investigated. This procedure has great promise as a therapeutic intervention that significantly reduces relapse in drug dependent clinical populations. However, it is clear that the retrieval – extinction procedure is more complicated than previously thought and it may, under some conditions, actually promote relapse. It is essential that we develop a better understanding of how modified extinction training protocols yield long lasting behavior change.

## Conflict of Interest Statement

The authors declare that the research was conducted in the absence of any commercial or financial relationships that could be construed as a potential conflict of interest.
